# Microstructural Evolution and Functional Response of a FeMnSiCr Shape Memory Alloy Module Processed by High-Speed High-Pressure Torsion

**DOI:** 10.3390/nano16211337

**Published:** 2026-10-22

**Authors:** Cristian Stefanescu, Carmela Gurau, Daniela-Laura Buruiana, Radu Ioachim Comăneci, Cosmin Romanitan, Gheorghe Gurau

**Affiliations:** 1 Interdisciplinary Research Centre in the Field of Eco-Nano Technology and Advance Materials CC-ITI, Faculty of Engineering, “Dunărea de Jos” University of Galati, 47 Domneasca Street, 800008 Galati, Romania; carmela.gurau@ugal.ro (C.G.); daniela.buruiana@ugal.ro (D.-L.B.); 2 Faculty of Materials Science and Engineering, “Gheorghe Asachi” Technical University of Iași, Bd. D. Mangeron 41, 700050 Iași, Romania; radu-ioachim.comaneci@academic.tuiasi.ro; 3 National Institute for Research and Development in Microtechnologies, 023573 Bucharest, Romania; cosmin.romanitan@imt.ro

**Keywords:** shape memory alloys, Fe-Mn-Si-Cr, severe plastic deformation, high-speed high-pressure torsion, martensitic transformation

## Abstract

In this study, a functional truncated-conical module made of the Fe57Mn27Si11Cr5 (at.%) shape memory alloy was investigated. The module was obtained by the severe plastic deformation process, High-Speed High-Pressure Torsion (HSHPT). It was processed in a single step, with the logarithmic strain estimated at *ε* = 1.10, and the correlation among the microstructure, structural state, and functional behavior was analyzed by optical microscopy (OM), scanning electron microscopy (SEM), X-ray diffraction (XRD), atomic force microscopy (AFM), and mechanical testing. HSHPT processing replaced the initial dendritic structure with a severely deformed and refined microstructure characterized by deformation bands, fibrous regions, and lamellar features associated with ε-martensite. XRD analysis revealed that γ-FCC austenite remained the dominant crystalline phase and that the γ(111) reflection shifted from 2θ = 43.47° to 43.12°. AFM analysis, performed over an area of 1600 µm^2^, revealed an arithmetic mean roughness (*R*_a_) of 14.59 nm and a root mean square roughness (*R*_q_) of 19.77 nm. Segmentation of the scanned area using the Flooding procedure revealed a mean Equivalent Circular Diameter (ECD) of 253.39 nm. Mechanical tests demonstrated stable functional behavior and progressive cyclic stabilization. The module exhibited a superelastic-like load–displacement response, accompanied by an increase in energy storage efficiency and a decrease in dissipated energy during repeated compression cycles.

## 1. Introduction

Iron-based shape memory alloys (Fe-SMAs) constitute an important class of functional metallic materials because they combine thermally activated shape recovery with the mechanical robustness, formability, and relatively low cost characteristic of Fe-based alloys. The scientific foundation of the Fe-Mn-Si family was established by the early demonstration of the shape memory effect associated with the stress-induced martensitic transformation from the face-centered cubic (γ) structure to the hexagonal close-packed (ε) structure, as well as by subsequent studies revealing its pronounced dependence on crystallographic orientation and alloy composition [1,2,3]. Unlike conventional shape memory systems based on thermoelastic transformations, the functional response of Fe–Mn–Si alloys is governed predominantly by the formation of deformation-induced ε-martensite and the reverse ε→γ transformation upon heating. The reversibility of this transformation and, consequently, the degree of shape recovery depend on the extent to which transformation strain can be accommodated without excessive activation of irreversible slip. Accordingly, review and mechanistic studies have emphasized that the shape memory response of Fe-based alloys cannot be separated from phase stability, defect structure, and deformation history [4,5].

The chemical composition of the alloy is essential to this balance. Mn, Si, and Cr influence austenite stability, stacking-fault energy (SFE), magnetic ordering, and the thermodynamic conditions of the γ↔ε transformation [6,7]. Si plays a particularly important role in increasing the reversibility of stress-induced martensite, whereas variations in Mn and Cr contents modify the relative stability of the FCC and HCP structures and the characteristic transformation temperatures [8,9,10]. The effects of other alloying elements confirm the same principle: C and Co, for example, can modify strengthening mechanisms and phase stability, thereby affecting shape memory performance [11]. Recent investigations comparing thermally induced and stress-induced martensite further show that the reverse ε→γ transformation is sensitive to the path by which martensite was generated [12]. These results establish composition, initial phase constitution, and deformation path as interdependent variables rather than independent material parameters.

The microstructural state is equally important. In polycrystalline Fe–Mn–Si alloys, incomplete recovery is associated with competition between reversible martensitic transformation and irreversible plastic deformation [13,14], while grain boundaries, dislocation structures, and local mechanical constraints influence the formation and reverse motion of ε martensite [15]. Consequently, numerous strategies have been developed to enhance recovery through control of the internal microstructure. Engineering of annealing twin boundaries can promote large recovery strains [16], whereas aligned second-phase particles and finely dispersed VC or NbC precipitates can enhance strengthening and modify transformation behavior [17,18,19,20,21,22]. Thermomechanical processing and training further reorganize the defect structure and modify the efficiency of subsequent transformation cycles [23,24].

The extensive microstructural and thermomechanical characterization reported for Fe57Mn27Si11Cr5 (at.%) thus provides a direct, material-specific context for the alloy investigated in the present work [25]. Previous studies on this material have shown that ausforming can improve the shape memory effect [26], while atomic force microscopy has revealed microstructural and surface-relief changes associated with training [27]. Grain size represents another critical variable: recovery stress and recovery strain evolve significantly over a broad grain-size range, and their response is correlated with annealing-twin density and the constraints imposed by grain boundaries [28,29].

The sensitivity of these mechanisms to microstructure provides a strong rationale for applying severe plastic deformation (SPD) to Fe-SMAs. Equal-channel angular pressing has demonstrated that very large strains can be combined with grain refinement and carbide precipitation in Fe-Mn-Si-Cr-Ni-C alloys [30]. More broadly, SPD processing can generate ultrafine-grained (UFG) or nanostructured states characterized by high dislocation densities, non-equilibrium grain boundaries, modified textures, and high defect-stored energies [31,32]. These effects are particularly important in shape memory alloys because the functional transformation itself is sensitive to interfaces, defects, and characteristic microstructural length scales [33]. High-pressure torsion (HPT), in particular, is one of the most effective SPD methods for introducing very large strains under quasi-hydrostatic pressure and has become a reference technique for producing bulk UFG and nanostructured metals [34]. In Fe-SMAs, however, grain refinement cannot be regarded as an isolated objective: the decisive question is whether the deformation-generated microstructure remains compatible with the γ↔ε transformation mechanisms required for functional recovery.

High-Speed High-Pressure Torsion (HSHPT) represents a development of severe plastic deformation processes in which high axial pressure is combined with torsional deformation produced at high rotational speeds, enabling large plastic strains to be introduced simultaneously with the forming of active elements having a predefined geometry. The application of HSHPT to Fe-Mn-Si-Cr shape memory alloys demonstrated the possibility of directly obtaining functional elements with rotational geometry [35]. Subsequent investigations revealed the formation of strongly refined or nanostructured microstructures in Fe-Mn-Si alloys after severe plastic deformation [36], as well as specific features of Fe-Mn-Si-Cr modules produced by HSHPT, including the formation and evolution of hardness gradients [37], superelastic-like mechanical responses [38], and the development of ultrafine-grained microstructures [39].

The functional behavior of Fe-Mn-Si alloys is determined by the interaction among mechanical loading, martensitic transformation, and the microstructural state of the material. Uniaxial deformation studies on Fe-Mn-Si-Cr-Ni-VC alloys have demonstrated the influence of the loading path on the evolution of the phase transformation [40], while recovery-stress measurements have shown the ability of these alloys to generate recovery forces with potential for engineering applications [41]. In situ neutron-diffraction investigations enabled direct correlation of strain evolution with phase transformations in Fe-Mn-Si alloys, while also revealing the influence of VC precipitates on material response [42]. Transfer to engineering applications also requires evaluation of material stability under service conditions, including corrosion behavior [43]. The functional properties of Fe-SMAs have led to the development of applications for strengthening and prestressing structures [44,45], a direction also reflected in recent reviews devoted to applications of these alloys [46]. The reversible γ↔ε transformation has also been exploited for vibration mitigation under cyclic tension–compression loading [47]. At the same time, recent research shows that functional properties can be tailored through control of microstructure and crystallographic texture, including by additive manufacturing [48], as well as through optimization of composition with respect to the targeted temperature range [49].

R3Q4-Although HSHPT processing of Fe-Mn-Si-Cr shape memory alloys and the feasibility of producing functional modules by this method have already been demonstrated, a comprehensive understanding of how the severe plastic deformation induced by HSHPT influences the resulting microstructure, phase constitution, and functional performance is still lacking. In particular, the relationship between the strongly refined microstructure, the phase transformations, and the thermomechanical response of HSHPT-processed functional modules has not yet been systematically established.

To address this gap, the present study investigates a Fe_57_Mn_27_Si_11_Cr_5_ (at.%) shape memory alloy processed by HSHPT into a truncated-conical functional module. The main objective is to establish correlations between the microstructural features, phase constitution, and functional behavior of the module. For this purpose, a multiscale characterization approach combining optical microscopy (OM), scanning electron microscopy (SEM), X-ray diffraction (XRD), and atomic force microscopy (AFM) was employed, while the functional response was evaluated through thermomechanical testing. By integrating these investigations, the study provides a detailed assessment of the microstructural and structural state generated by HSHPT processing and its influence on the functional performance of the Fe_57_Mn_27_Si_11_Cr_5_ module. The results contribute to a better understanding of structure-property relationships in HSHPT-processed Fe-Mn-Si-Cr alloys and provide an experimental basis for the future development of shape memory alloy-based composites and functional components.

## 2. Materials and Methods

**Figure 1:**
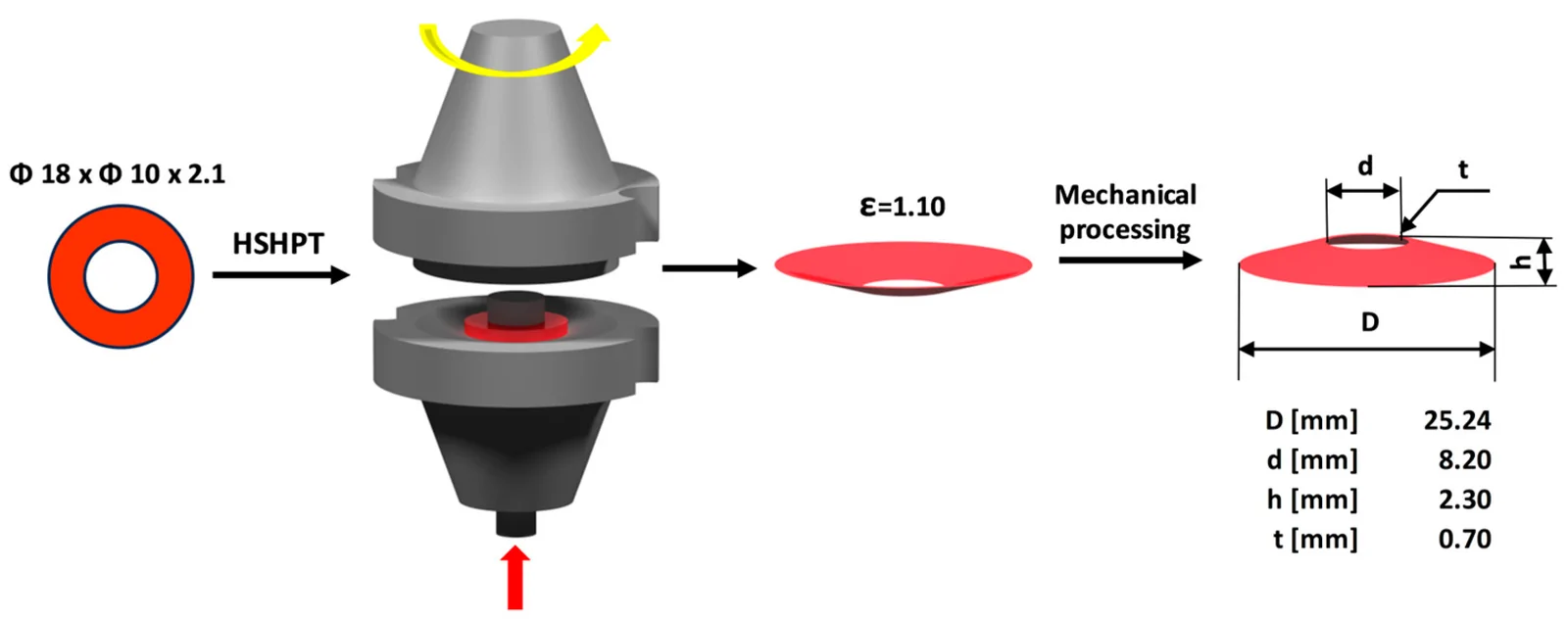
Processing route of the Fe57Mn27Si11Cr5 SMA module.

For this study, a truncated-conical module made of the Fe57Mn27Si11Cr5 (at.%) shape memory alloy was fabricated by the High-Speed High-Pressure Torsion (HSHPT) severe plastic deformation method. A schematic representation of the manufacturing process is illustrated in Figure 1. The sample was obtained from a ϕ20 × 30 mm ingot produced by cold-crucible induction melting from high-purity elemental powders (>99%) using a Fives Celes MP25 furnace (Lautenbach, France). The nominal alloy composition used for preparation, expressed in weight percent, was 61% Fe, 28% Mn, 6% Si, and 5% Cr. Following preliminary machining and axial drilling (ϕ10 mm), the ingot was sectioned using a low-speed diamond saw into rings with dimensions of ϕ18 × ϕ10 × 2.1 mm.

The true strain attained in the processed material was estimated using the following relationship [34]:

(1) $$\varepsilon =\ln\frac{h_{i}}{h_{f}}$$

where *h*_i_ and *h*_f_ represent the initial and final sample thicknesses, respectively. According to this relationship, the calculated true strain was *ε* = 1.10.

The Fe-Mn-Si-Cr module is geometrically characterized by the outer diameter (*D*), inner diameter (*d*), initial height (*h*), and wall thickness (*t*). Based on the geometrical characteristics of conical active elements [50], the characteristic shape ratio (SCR) was determined using the following relationship:

(2) $$\mathrm{SCR}=\frac{h-\;t}{t}$$

The geometrical parameters of the Fe-Mn-Si-Cr module are presented in Table 1.

Samples intended for microstructural investigations via optical microscopy (OM), scanning electron microscopy (SEM) and atomic force microscopy (AFM) were prepared by diametrical sectioning using an Accutom-100 machine (Struers GmbH, Willich, Germany), followed by hot mounting in PolyFast (Struers GmbH, Willich, Germany) conductive resin on a CitoPress-30 (Struers GmbH, Willich, Germany) system and subsequent grinding/polishing on a Tegramin-30 machine (Struers GmbH, Willich, Germany), in accordance with standard metallographic procedures at the Struers, Bucharest, Romania Laboratory.

To reveal the microstructure, the surfaces of the Fe-Mn-Si-Cr samples were subsequently chemically etched in a solution consisting of 1.2 g K_2_S_2_O_5_ and 1 g NH_4_HF_2_ dissolved in 100 mL of distilled water.

Comprehensive details regarding the HSHPT procedure and the custom-built apparatus employed in this study have been extensively documented in our previous publications [35,51].

Optical microscopy investigations were performed using an OLYMPUS BX51 microscope (Tokyo, Japan), equipped with a digital camera controlled via QuickPHOTO MICRO 2.3 software.

SEM micrographs were obtained using a FEI Quanta 200 3D DualBeam microscope (Hillsboro, OR, USA) at an accelerating voltage of 20 kV.

The X-ray diffraction (XRD) patterns were acquired using a 9 kW Rigaku SmartLab diffractometer (Osaka, Japan) equipped with a Cu Kα1 source (λ = 0.15406 nm). The θ–2θ range was scanned from 10 to 90° with a step size of 0.01° and a scan rate of 5°/min.

The roughness parameters, such as the root-mean-square (RMS) roughness and the arithmetic average roughness (*R*_a_), were evaluated via atomic force microscopy (AFM) measurements performed using a Nanonics MultiView 4000 system, (Nanonics Imaging Ltd., Jerusalem, Israel) in intermittent mode. AFM measurements were performed over a scanning area of 40 × 40 µm^2^. The analysis was conducted with 20 nm diameter probes, a vibration frequency of 37 kHz, and a quality factor (*Q*) of ~1900. The AFM analysis data were processed by means of the WSxM 5.0 Develop 12.0 software to obtain the 3D roughness maps, the corresponding statistical roughness distributions, and the equivalent circle diameter (ECD) of the refined grains [52].

The mechanical testing was conducted on an Instron 3382 (Instron, Norwood, MA, USA) universal testing machine (capacity 100 kN) with an Instron 3119-506 environmental chamber for investigations in the temperature range of −70 to +350 °C. The temperature was controlled by a Eurotherm 2100P controller (Instron, Norwood, MA, USA) with an accuracy of 0.2%, and the load measurement accuracy was 0.2%. The resolution of the crosshead position was 0.0598 µm, and the displacement measurement accuracy was 0.5%. The Bluehill Series IX™ software (version 4.55) was used for data acquisition and control of the parameters (load, displacement, strain rate, and temperature). Moreover, the load cell was thermally insulated by a ceramic coating to minimize heat transfer to the force transducer and reduce thermally induced measurement effects during long-term experiments. Two different testing protocols were applied to the Fe–Mn–Si–Cr module. In the first protocol, the sample was subjected to five static compression cycles up to a stroke of 1 mm between flat platens at room temperature, using a constant crosshead speed of 0.5 mm/min, to evaluate the load–stroke response. The second testing protocol involved compressing the module at a constant stroke of 0.4 mm, followed by heating to 180 °C in a fully constrained state to demonstrate the shape memory effect (SME) under constrained recovery conditions and to evaluate the generated recovery force.

## 3. Results and Discussion

### 3.1. Severe Plastic Deformation via High-Speed High-Pressure Torsion

**Figure 2:**
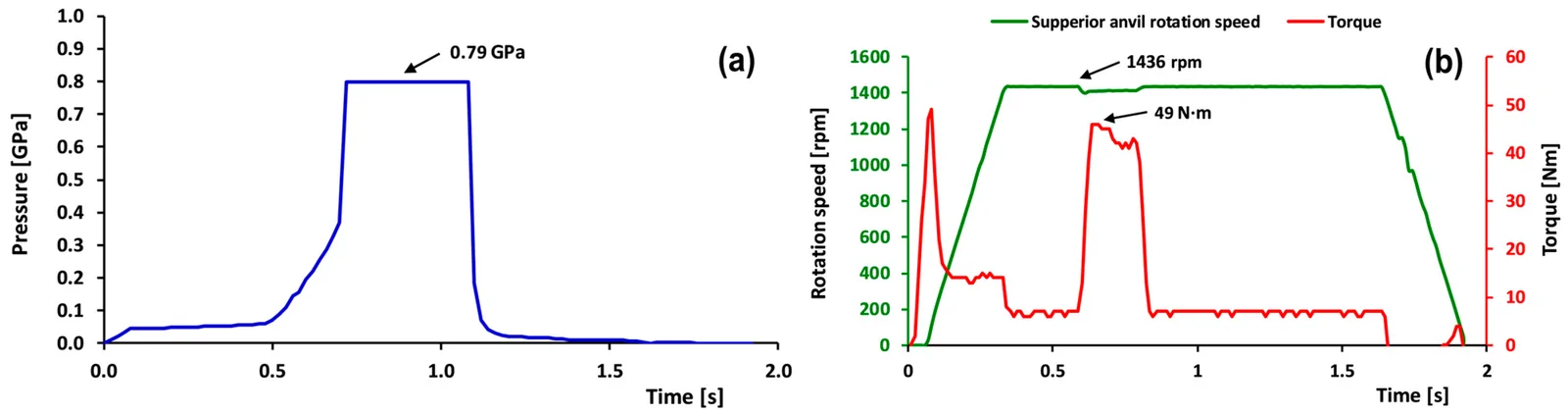
Variation in the HSHPT processing parameters versus time: (**a**) pressure evolution during HSHPT processing; (**b**) evolution of the rotational speed and torque during HSHPT processing.

As illustrated in Figure 1, the Fe-Mn-Si-Cr shape memory module was fabricated in a single HSHPT processing step. The Fe-Mn-Si-Cr circular ring, with dimensions of ϕ18 × ϕ10 × 2.1 mm, was positioned on the centering pin of the lower anvil, while the truncated-conical geometry of the module was imposed by the specific configuration of the anvils. The HSHPT process lasted approximately 2 s. The maximum recorded quasi-hydrostatic pressure applied to the lower punch was 0.79 GPa (Figure 2a), while the upper punch operated at a rotational speed of 1436 rpm (Figure 2b). The torque measured on the motor shaft during severe plastic deformation exhibited two distinct peaks. The first peak was associated with the breakaway of the ring from its initial sticking condition, after which the ring began to rotate in contact with the upper anvil. The second peak occurred during severe plastic deformation and reached a torque value of 49 Nm (Figure 2b). The rotational speed of the upper punch exhibited a slightly perturbed plateau because part of the available power was absorbed during the SPD process (Figure 2b). Finally, a specially designed mandrel was used to machine the outer diameter of the module by turning, with the final dimensions reported in Table 1.

### 3.2. Optical Microscopy

**Figure 3:**
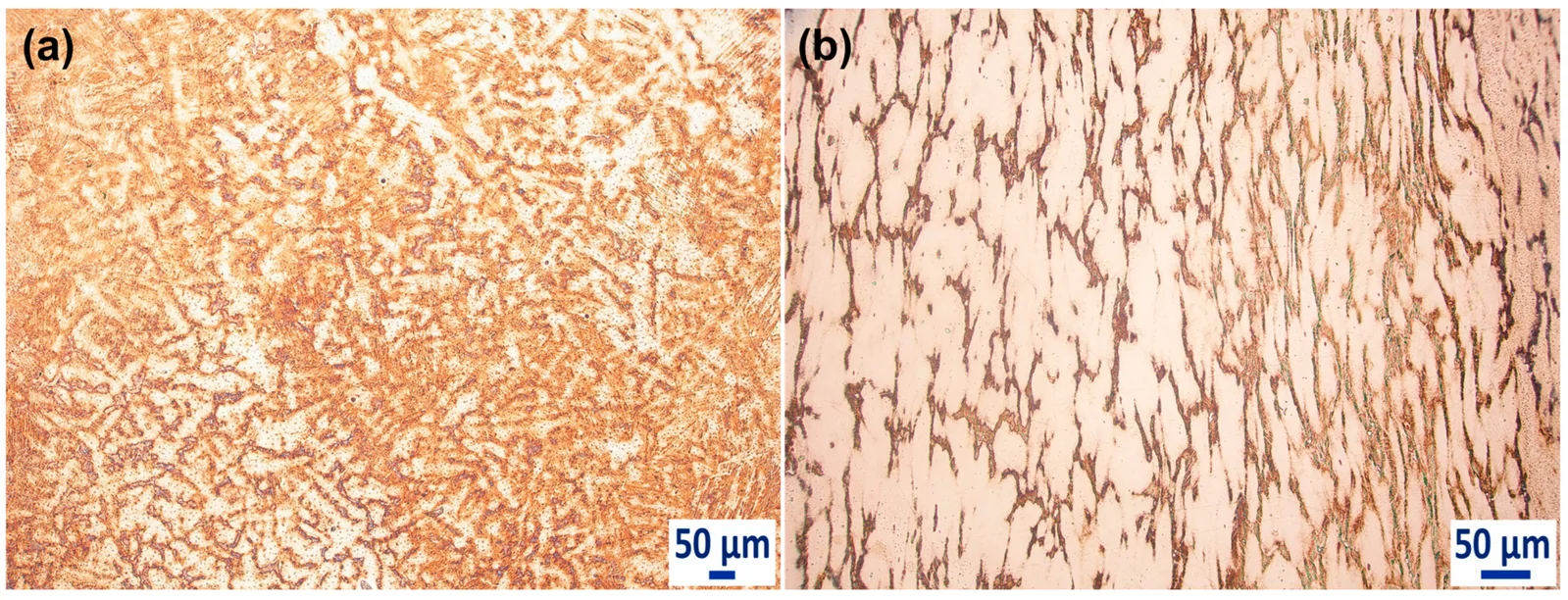
Optical micrographs: (**a**) Fe-Mn-Si-Cr as-received; (**b**) Fe-Mn-Si-Cr module (*ε* = 1.10).

The microstructure was investigated in the initial sample and in the truncated-conical module fabricated by HSHPT. The microstructure of the Fe-Mn-Si-Cr alloy in the as-received state exhibits a dendritic morphology characteristic of solidification processes, consisting of γ-austenite (FCC) dendrites and interdendritic regions. The coarse structure and heterogeneous distribution of the microstructural constituents are characteristic of the material prior to severe plastic deformation processing and represent the starting point for the subsequent processes of crystalline grain fragmentation and structural refinement (Figure 3a) [34,35,53].

After HSHPT processing to a logarithmic strain of *ε* = 1.10, the initial dendritic structure is completely eliminated and replaced by a severely deformed microstructure. The microstructure of the truncated-conical module is characterized by deformation bands and fibrous regions preferentially oriented along the material flow direction (Figure 3b). The observed appearance indicates fragmentation of the γ-austenite grains and the development of a refined structure under the combined effect of hydrostatic compression and torsional shear [34,35,53,54,55]. The optical micrographs reveal the elimination of the initial dendritic morphology and the formation of deformation bands and elongated fibrous regions associated with material flow during HSHPT processing. Because optical microscopy does not provide sufficient spatial resolution for direct observation of nanoscale defects, stacking faults, partial dislocations, and dislocation arrangements were not directly characterized in the present study. The observed morphology is nevertheless consistent with the structural refinement commonly reported for Fe-Mn-Si-Cr alloys subjected to severe plastic deformation. This observation is consistent with results reported for Fe-Mn-Si-Cr alloys after HSHPT processing, in which the dendritic structure is replaced by a continuously fibrous and severely deformed microstructure [35,54].

### 3.3. SEM Results

**Figure 4:**
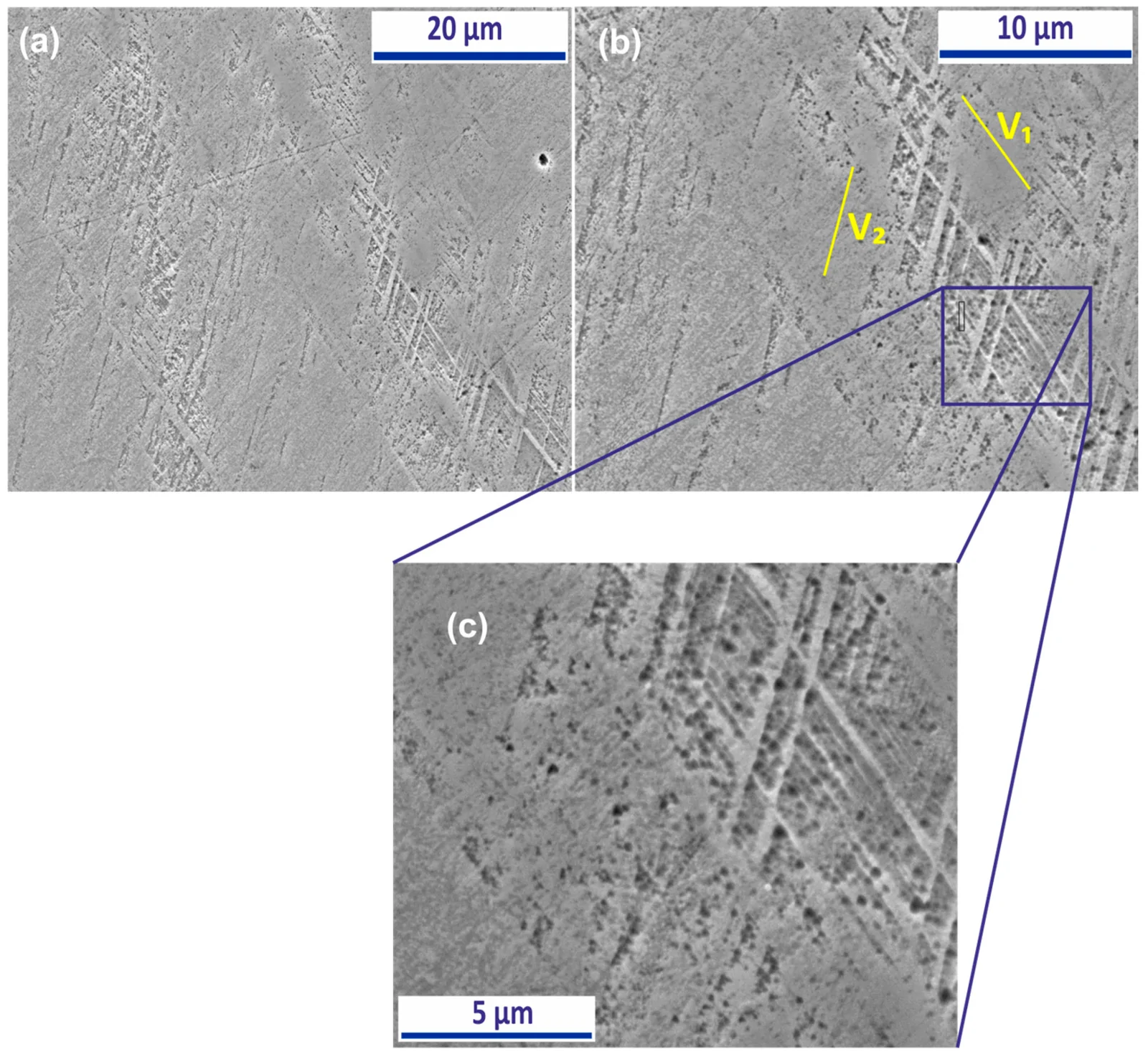
SEM micrographs of the Fe-Mn-Si-Cr module after HSHPT processing: (**a**) general view of the deformed microstructure; (**b**) higher-magnification detail showing the lamellar regions and the orientations of variants 1 and 2 of the ε-HCP martensite plates; (**c**) fine plates of stress-induced ε-martensite.

The SEM image of the truncated-conical module obtained by HSHPT (Figure 4a) shows a strongly refined microstructure consisting of deformation bands and regions with a contrast compatible with the deformation-induced martensitic transformation.

The SEM micrographs reveal deformation bands, lamellar contrast features, and strongly deformed regions, indicating significant structural modification after HSHPT processing. While SEM observations do not allow the direct identification of stacking faults, individual dislocations, or dislocation networks, the interpretation of stacking-fault accumulation and dislocation generation is supported both by deformation mechanisms extensively reported in the literature for Fe-Mn-Si-Cr alloys subjected to severe plastic deformation and by our previously published results, which provide experimental evidence for the operation of these mechanisms under similar processing conditions. At the applied strain of *ε* = 1.10, the high density of dislocations and stacking faults promotes the nucleation of ε-martensite in the austenitic matrix. In Fe-Mn-Si-Cr alloys with low stacking-fault energy (SFE), stacking faults and partial dislocations accumulate and induce the local transformation of γ-austenite (FCC) into ε-martensite (HCP). The observed morphology indicates coexistence of the γ and ε phases, which is necessary for the manifestation of the shape memory effect [14,54].

At higher magnification, lamellar regions, a deformation-induced texture, and two preferred orientations of the ε-HCP martensite variants (V1 and V2) can be distinguished, associated with the strongly deformed microstructure generated during HSHPT processing (Figure 4b). At the applied strain, *ε* = 1.10, only two of the three possible variants were identified in the SEM micrographs. Therefore, the refined structure obtained by HSHPT changes the triangular morphology typical of ε-martensite, indicating that not all three variants were observed under the processing conditions used [34,54,55]. The literature indicates that dislocation activity and stacking-fault formation can contribute to the nucleation and stabilization of ε-martensite in Fe-Mn-Si-Cr alloys. In the present study, these mechanisms are discussed as possible interpretations supported by previous investigations [34,54,55,56].

Recent studies on Fe-Mn-Si alloys after HPT have shown that structural refinement is accompanied by an increase in the martensitic fraction and stabilization of the γ→ε transformation [57,58]. Recent literature indicates that the accumulation of stacking faults and internal stresses represents the principal mechanism for stabilizing ε-martensite in Fe-Mn-Si alloys processed by severe plastic deformation [56,57,58].

The higher-magnification SEM micrograph (Figure 4c) reveals a highly refined microstructure characterized by lamellar features and severely deformed regions, indicating extensive microstructural evolution induced by HSHPT processing. Although the SEM observations provide clear evidence of significant structural refinement, they do not enable direct quantification of the nanoscale grain size or direct assessment of the dislocation density.

The reduced size of the structural features and the high degree of fragmentation observed indicate an advanced level of microstructural refinement produced by severe plastic deformation (SPD) [34,53,55,56]. This combination of crystalline grain refinement, stacking-fault accumulation, and ε-martensite stabilization is considered one of the main factors responsible for the improvement in functional behavior and the increased reversibility of the martensitic transformation in Fe-Mn-Si-Cr alloys following HSHPT deformation [14,54,57].

### 3.4. X-Ray Diffraction (XRD)

**Figure 5:**
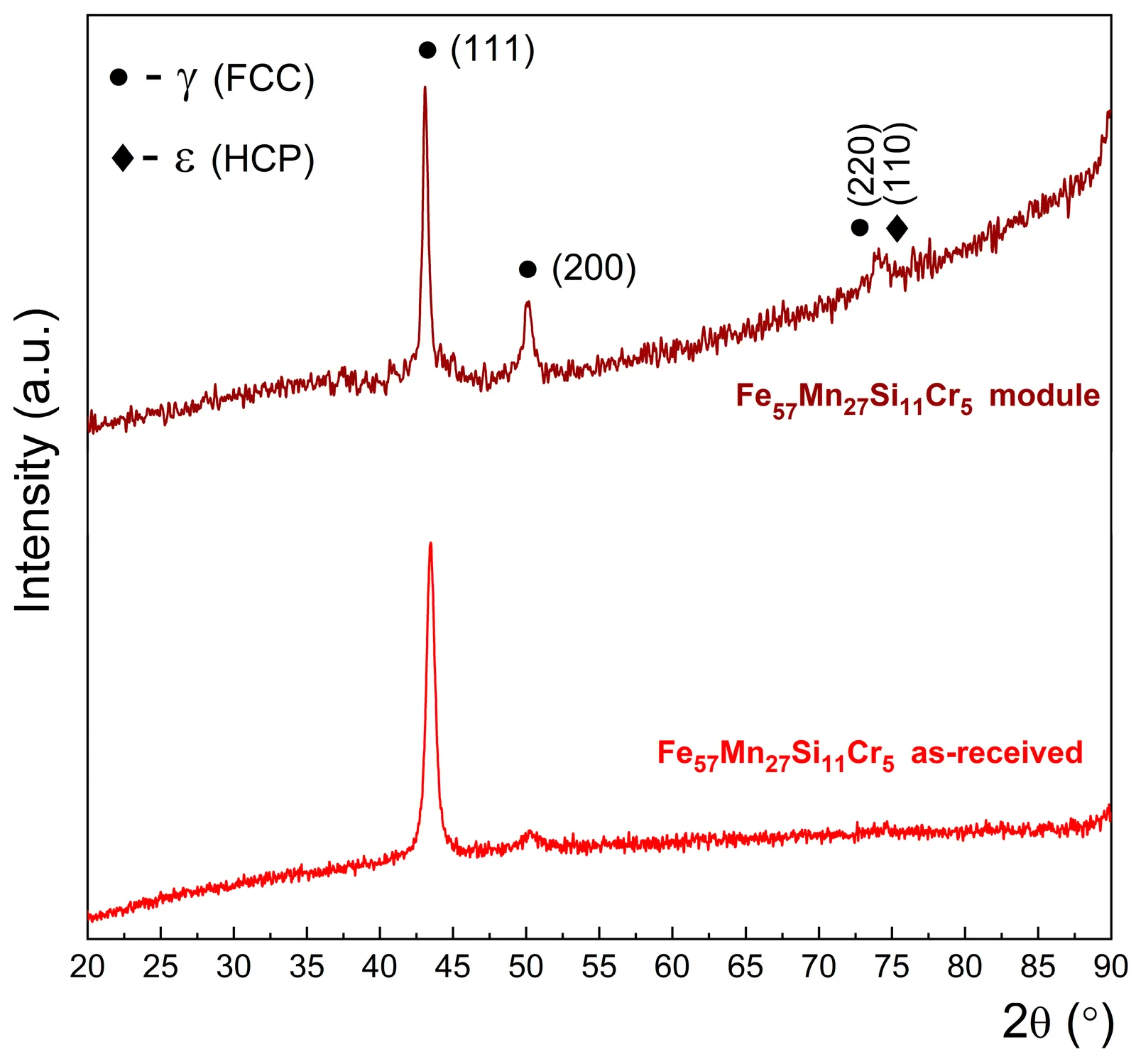
X-ray diffraction (XRD) patterns of the Fe-Mn-Si-Cr module and the Fe-Mn-Si-Cr alloy in the as-received state.

The X-ray diffraction (XRD) patterns of the Fe-Mn-Si-Cr alloy as-received and the Fe-Mn-Si-Cr module processed by HSHPT are presented in Figure 5. The comparative XRD patterns of the Fe57Mn27Si11Cr5 SMA alloy as-received and of the module after severe plastic deformation reveal the predominance of the γ-austenite phase with a face-centered cubic (FCC) structure [59].

Although lamellar features compatible with deformation-induced ε-martensite were observed, only one diffraction peak, corresponding to the ε-HCP (110) plane, could be identified. The absence of additional characteristic ε-HCP reflections may be attributed to the low volume fraction of ε-martensite induced by HSHPT.

At the same time, the γ (220) reflection, which is not distinctly evident in the XRD pattern of the alloy in the as-received state, becomes evident after HSHPT processing. This evolution indicates a modification of the diffraction profile of the γ-FCC phase after severe plastic deformation, without being ascribed to a single mechanism. For quantitative evaluation of the structural changes, the main γ (111) reflection was analyzed separately by nonlinear fitting using the Voigt function in Origin, with the results presented in Table 2. The fitting yielded peak centers at 2θ = 43.47° for the initial alloy and 43.12° for the Fe-Mn-Si-Cr module, with adjusted *R*^2^ values of 0.998 and 0.990, respectively. The shift of Δ2θ = −0.36° toward lower angles after HSHPT processing indicates an increase in the interplanar spacing *d*_111_ from approximately 2.08 Å to 2.10 Å.

The FCC lattice parameter, calculated from the γ(111) reflection, correspondingly increases from approximately 3.60 Å to 3.63 Å. This trend indicates a change in the crystal lattice state after severe plastic deformation and is consistent with lattice distortions and residual stresses introduced by HSHPT, without attributing the peak shift to a single mechanism.

The mean coherent diffraction domain size, *τ*, was estimated using the Scherrer equation:

(3) $$\tau =\frac{K\cdot \lambda }{\beta \cdot \cos\theta }$$

A shape factor of *K* = 0.939 was adopted, corresponding to cubic crystallite geometry when peak width is expressed as FWHM (Full Width at Half Maximum) [60]. For Cu Kα radiation, *λ* = 1.5406 Å was used. The Voigt parameters yielded FWHM values of approximately 0.69° for the initial alloy and 0.45° for the Fe-Mn-Si-Cr module, resulting in *τ* ≈ 12.93 nm and *τ* ≈ 20.02 nm, respectively. The *τ* values represent the size of coherently diffracting crystalline domains. Because the *τ* values were calculated without correction for instrumental broadening, they are regarded as comparative estimates of the coherent domain size [60,61]. Microstrain was not evaluated separately in the Scherrer calculation. The decrease in the FWHM of the γ(111) reflection and the corresponding increase in the Scherrer value are not interpreted, when considered separately, as direct evidence of a physical increase in coherent domain size after HSHPT processing. For evaluation of the microstrain contribution, the Williamson–Hall relation was used:

(4) $$\beta \cdot \cos\theta =\frac{K\cdot \lambda }{\tau }+4\cdot \varepsilon \cdot \sin\theta$$

**Figure 6:**
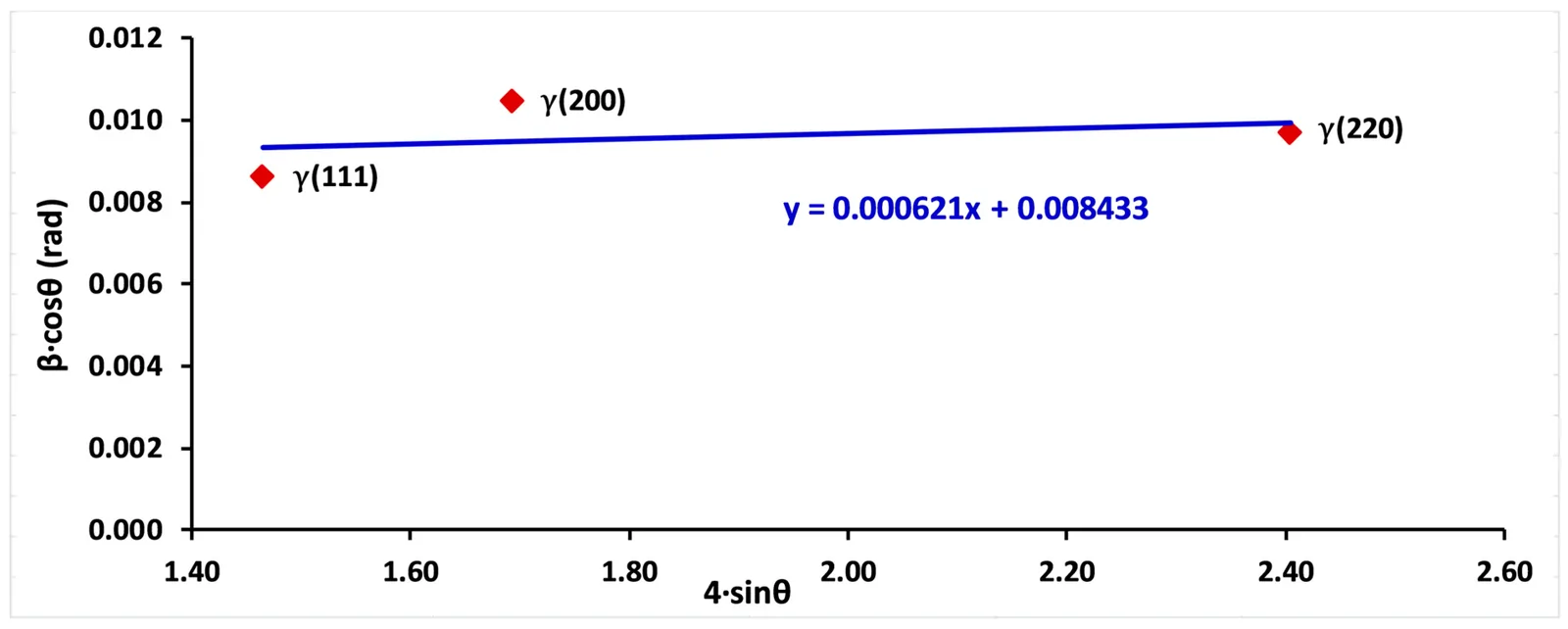
Williamson–Hall plot for the γ-FCC phase of the Fe-Mn-Si-Cr module after HSHPT, based on the γ(111), γ(200), and γ(220) reflections.

Williamson–Hall analysis of the γ(111), γ(200), and γ(220) reflections of the Fe-Mn-Si-Cr module after HSHPT (Figure 6) yielded a mean coherent diffraction domain size of *τ* ≈ 17.16 nm and a microstrain of *ε* ≈ 0.062%. Given the low linear correlation (*R*^2^ = 0.107), these values are considered complementary estimates of the structural state of the γ-FCC phase. The higher *β*cos*θ* value observed for the γ(200) reflection may be associated with anisotropic diffraction peak broadening, arising from crystallographic orientation-dependent variations in coherent diffraction domain size and microstrain. This deviation from the linear Williamson–Hall trend is consistent with the low coefficient of determination.

The comparative interpretation of the XRD results is based mainly on the reflection positions and the structural parameters determined by fitting. Overall, the XRD results indicate that HSHPT processing retained γ-FCC austenite as the dominant crystalline phase while producing measurable changes in its structural state.

### 3.5. Atomic Force Microscopy (AFM)

AFM analysis was used for the quantitative characterization of surface topography and of the grain-size distribution identified on the surface of the Fe57Mn27Si11Cr5 (at.%) module processed by HSHPT. In accordance with reporting practices commonly used in AFM studies focused on morphological characterization, the interpretation was developed starting from the topographic maps, followed by roughness parameters and, separately, by grain segmentation and statistical analysis. WSxM 5.0 Develop 12.0 software enables both calculation of surface statistical parameters and analysis using the Flooding procedure [52], while recent AFM characterization approaches emphasize the usefulness of combining topographic information with quantitative surface descriptors [62]. It should be noted that AFM topographic measurements characterize surface morphology rather than the underlying crystallographic structure. Therefore, the segmented features identified using the Flooding procedure cannot be unequivocally considered individual crystallographic grains. Consequently, the obtained ECD values should be interpreted as characteristic dimensions of the segmented topographic features under the applied analysis conditions rather than as direct measurements of grain size.

**Figure 7:**
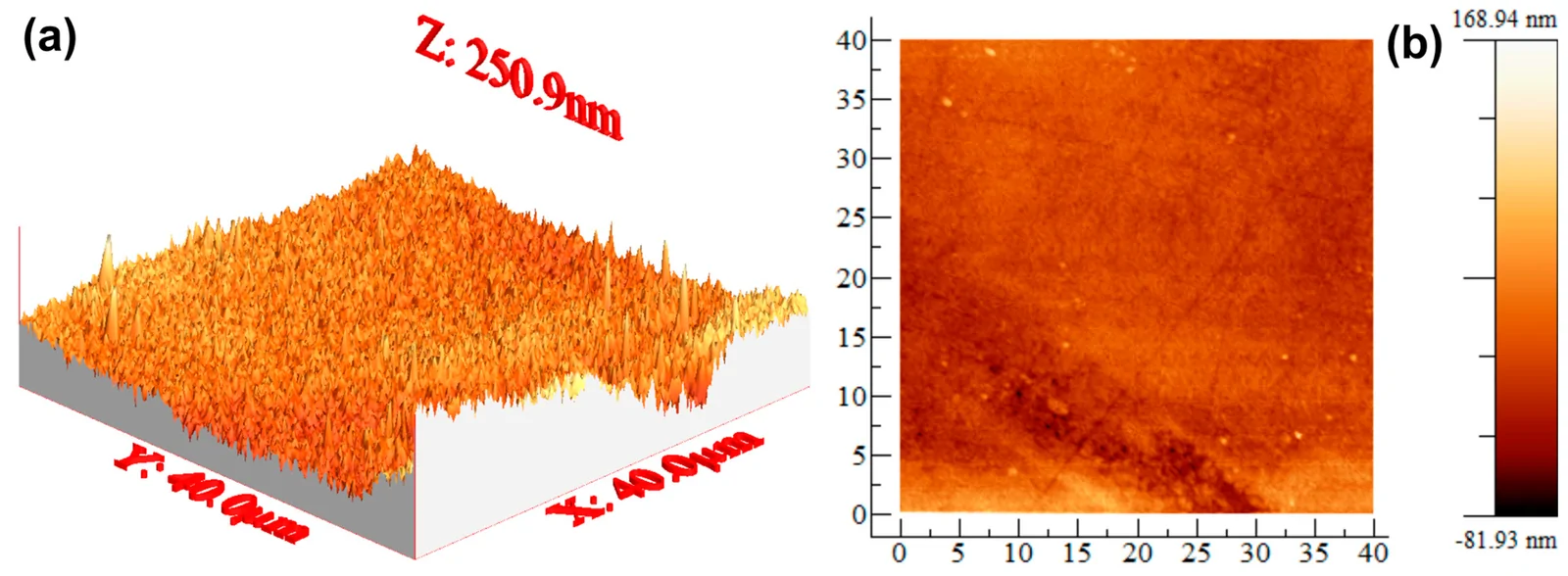
AFM topographic maps of the surface of the Fe57Mn27Si11Cr5 module, following HSHPT deformation acquired over a 40 × 40 µm^2^ scan area: (**a**) three-dimensional (3D) representation; (**b**) two-dimensional (2D) representation.

The 3D and 2D maps in Figure 7 reveal a fine but non-uniform topography, characterized by numerous asperities distributed over the entire scanned surface and by a limited number of more pronounced protrusions. The extreme values of the vertical coordinate are −81.93 nm and 168.94 nm, resulting in a peak-to-peak range of 250.87 nm, in agreement with the vertical range of approximately 250.9 nm indicated by the 3D map. Therefore, the surface is not characterized by uniform height variations but rather by a relatively fine background topography on which more pronounced local asperities are superimposed.

The parameter values listed in Table 3 support the topographic observations. The arithmetic mean roughness is *R*_a_ = 14.59 nm, while the root-mean-square roughness is *R*_q_ = 19.77 nm, giving an *R*_q_/*R*_a_ ratio of approximately 1.35. Because *R*_q_ gives greater weight to high-amplitude deviations, the difference between *R*_q_ and *R*_a_ is consistent with the presence of the local protrusions visible in the 3D map. The height distribution is positively skewed, as indicated by *S*_sk_ = 0.80, whereas *S*_ku_ = 6.08, a value greater than 3, indicates a leptokurtic distribution in which extreme topographic deviations contribute more strongly than in a normal distribution. Consequently, *R*_a_ and *R*_q_ describe the overall roughness level, whereas *S*_sk_ and *S*_ku_ confirm the asymmetric and pronounced character of the surface asperities. The combined treatment of morphology and quantitative roughness descriptors is also used in recent AFM studies of surface characterization [62].

For grain-size analysis, the AFM image was processed using WSxM 5.0 Develop 12.0 software through the Flooding procedure. The 2D image was analyzed using the following segmentation parameters: Minimum Grain Area = 0.035 µm^2^ and Minimum Height = 30 nm. Holes were excluded from the analysis so that the statistical interpretation was performed only on the grains identified on the scanned surface. In WSxM, Flooding is a procedure intended for the separation and quantification of topographic features based on a user-defined threshold, allowing the software to calculate the area, perimeter, and other statistical quantities of the resulting regions [52]. The use of the Flooding function for quantifying features in AFM images has also been reported by other authors, for example, for evaluating surface areas and populations of islands/grains [63,64].

**Figure 8:**
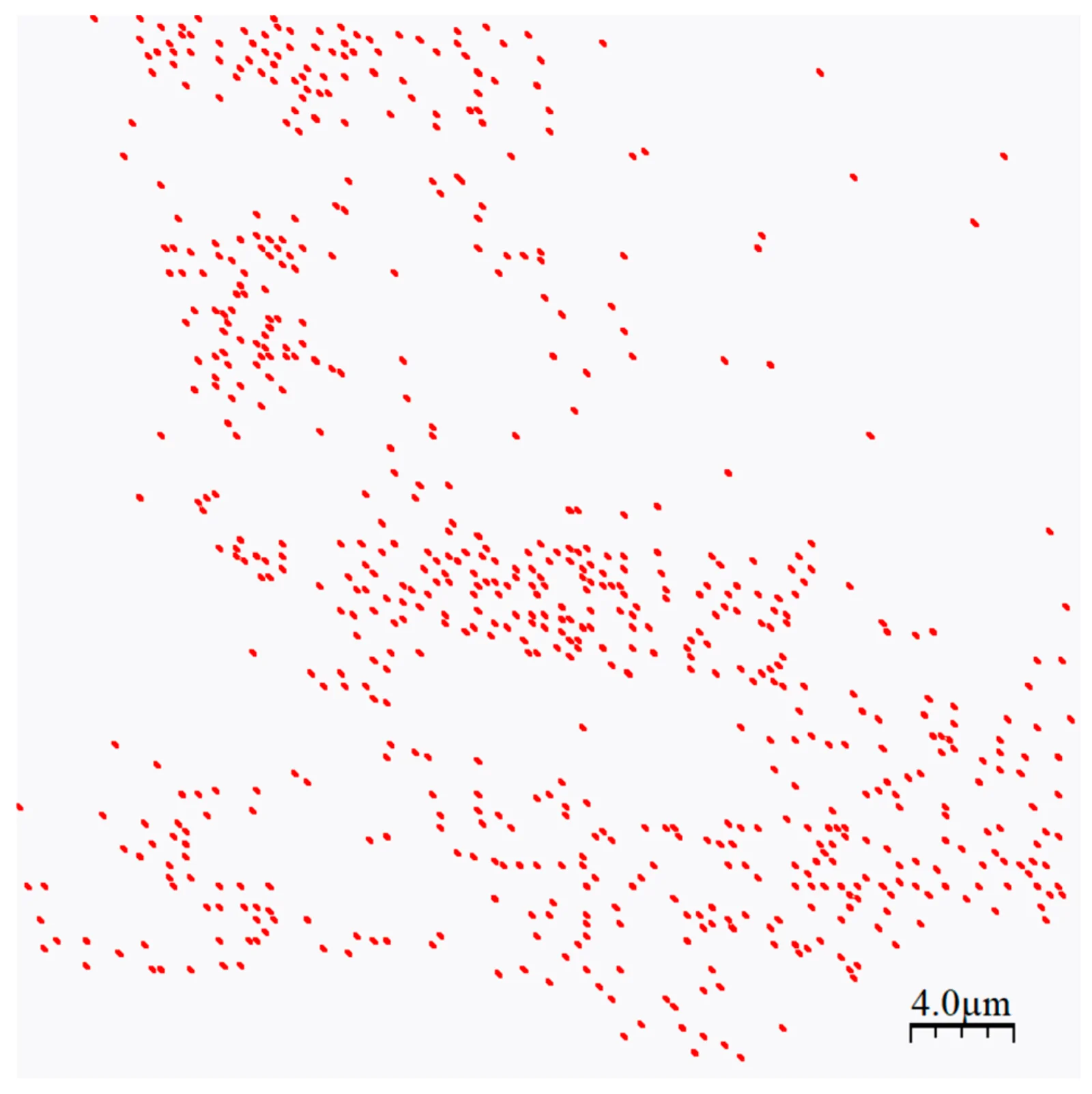
Grain map obtained after applying the Flooding procedure in WSxM to the 40 × 40 µm^2^ AFM surface.

Flooding The Flooding procedure (Figure 8) reveals the distribution of the grains identified over the entire scanned surface. Segmentation resulted in the identification of *N* = 677 grains. The spatial distribution is non-uniform, with regions in which the grains are more densely grouped and regions with lower grain density. The non-uniform distribution of the grains identified using the Flooding procedure may be associated with local variations in surface topography. Regions with a higher concentration of red markers correspond to areas with a higher density of grains meeting the applied segmentation criteria, whereas unmarked regions indicate that no grains were detected under the applied analysis conditions, without implying the absence of crystallographic grains. Because the purpose of the procedure is the statistical interpretation of the segmented topography, the map is used here as the basis for dimensional quantification of the grain population, without assigning to contrast variations any significance beyond that derived from the AFM analysis. The size of each grain was expressed in terms of the Equivalent Circular Diameter (ECD), defined as the diameter of a circle having the same projected area as the segmented grain. For a grain with projected area *A*, the ECD was calculated according to the following relationship:

(5) $$\mathrm{ECD}=2\sqrt{\frac{A}{\pi }}$$

**Figure 9:**
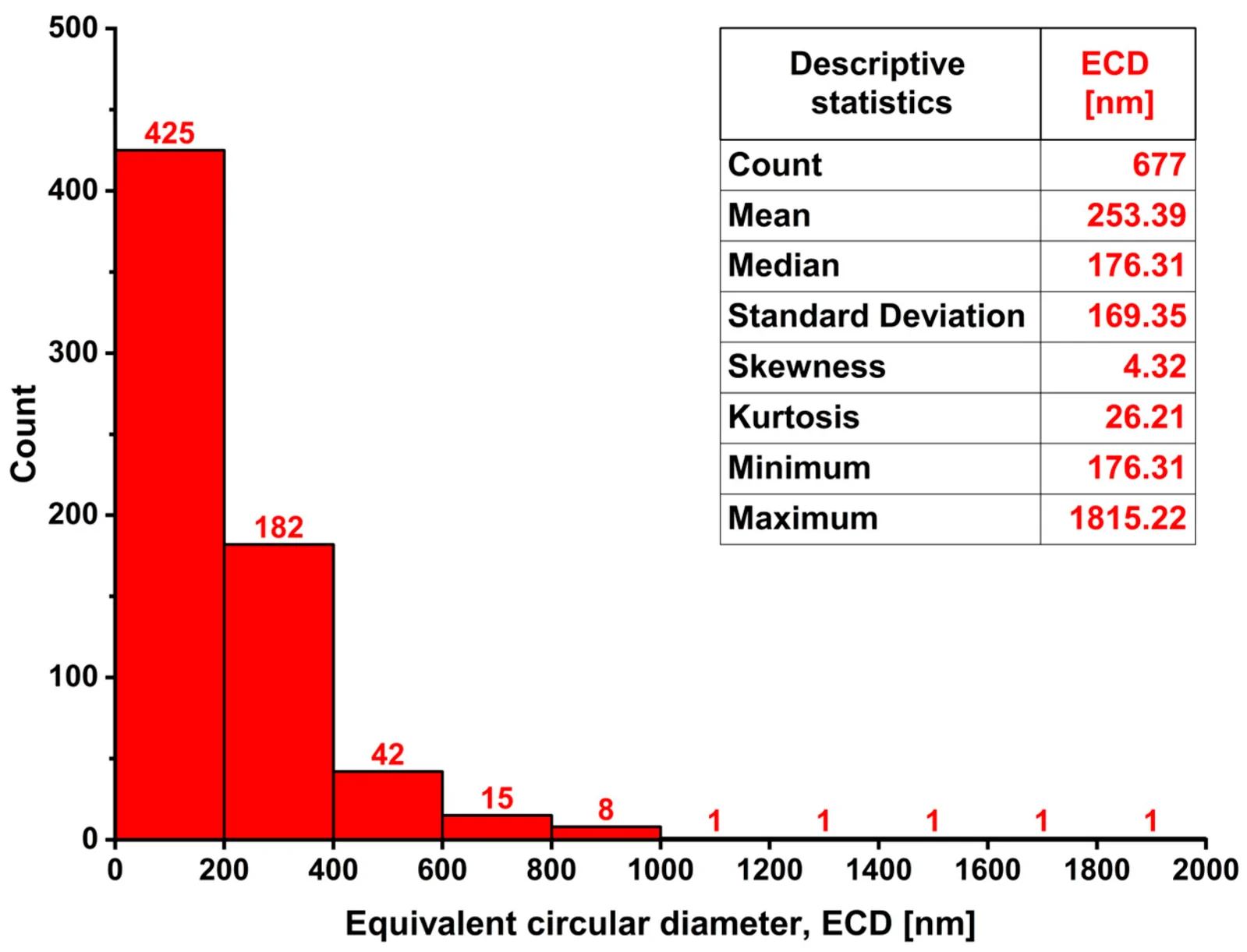
Histogram of the equivalent circular diameter (ECD) distribution of the grains identified by the Flooding procedure and the corresponding statistical parameters (bin width = 200 nm).

The equivalent circular diameter reduces an irregular planar geometry to a single, comparable dimensional parameter without assuming that the grain is actually circular. This reporting approach is commonly used in quantitative analyses of microstructures and segmented features, including studies in which grain or island size is quantified through the diameter of a circle with equivalent area [64]. The ECD histogram (Figure 9) and the statistical parameters show that the size distribution is strongly skewed toward larger values. The mean ECD is 253.39 nm, whereas the median is 176.31 nm, and the standard deviation of 169.35 nm indicates a broad size dispersion. The difference between the mean and the median results from the tail of the distribution toward larger sizes and is confirmed by the skewness value of 4.32. The very high kurtosis value of 26.21 indicates a strongly leptokurtic distribution, in which most measurements are concentrated in the lower size range while a limited number of much larger grains are also present. This behavior is further illustrated by the difference between the minimum value of 176.31 nm and the maximum value of 1815.22 nm.

Analysis by size classes provides a more representative picture than the mean value considered alone. A total of 425 grains, representing approximately 62.8% of the population, fall within the ECD < 200 nm class, whereas 182 grains lie between 200 and 400 nm. Thus, 607 of the 677 grains, corresponding to approximately 89.7% of the analyzed population, have ECD values below 400 nm. The number of grains decreases rapidly in the higher size classes: 42 grains are between 400 and 600 nm, 15 between 600 and 800 nm, and 8 between 800 and 1000 nm; only five grains exceed 1 µm. The distribution is therefore numerically dominated by submicrometric grains, while the small fraction of larger grains shifts the mean toward higher values.

A particular feature of the distribution is the coincidence between the minimum and median ECD (176.31 nm), associated with the pronounced accumulation of measurements at the lower end of the distribution. This value represents the lower threshold for individual grain quantification under the AFM analysis conditions used and not the minimum physical grain size in the material. Consequently, the microstructural population may include grains smaller than 176.31 nm that could not be individually quantified by the ECD analysis performed over the 40 × 40 µm^2^ scan area. The accumulation of 62.8% of the ECD values in the first size class indicates a substantial concentration of the population toward the lower quantification limit and is compatible with the presence of a significant fraction of grains below this threshold. Therefore, the measured ECD distribution predominantly characterizes the UFG size range accessible to AFM quantification, without excluding the presence of a population of nanoscale grains below the 176.31 nm limit. Studies on automated SPM image analysis have shown that segmentation results can be sensitive to image resolution and the identification procedure, supporting the use of constant settings for statistical comparison and a cautious interpretation of the smallest-size population [65].

Overall, the AFM analysis describes a surface with moderate nanoscale roughness, a height distribution dominated by localized positive asperities, and a predominantly submicrometric grain population. *R*_a_ = 14.59 nm and *R*_q_ = 19.77 nm characterize the overall roughness level, whereas *S*_sk_ = 0.80 and *S*_ku_ = 6.08 reveal the asymmetry and leptokurtic character of the topographic distribution. Independently, the Flooding analysis identifies 677 grains and reveals a strongly right-skewed ECD distribution, in which 89.7% of the grains have ECD < 400 nm, with a mean value of 253.39 nm. Thus, the roughness descriptors, Flooding map, and ECD statistics together provide a coherent and strictly quantitative AFM characterization of the analyzed surface. The results indicate a predominantly ultrafine microstructure within the size range quantifiable by AFM, together with evidence for a population of nanoscale grains below the lower ECD quantification threshold of 176.31 nm.

### 3.6. Mechanical Tests

**Figure 10:**
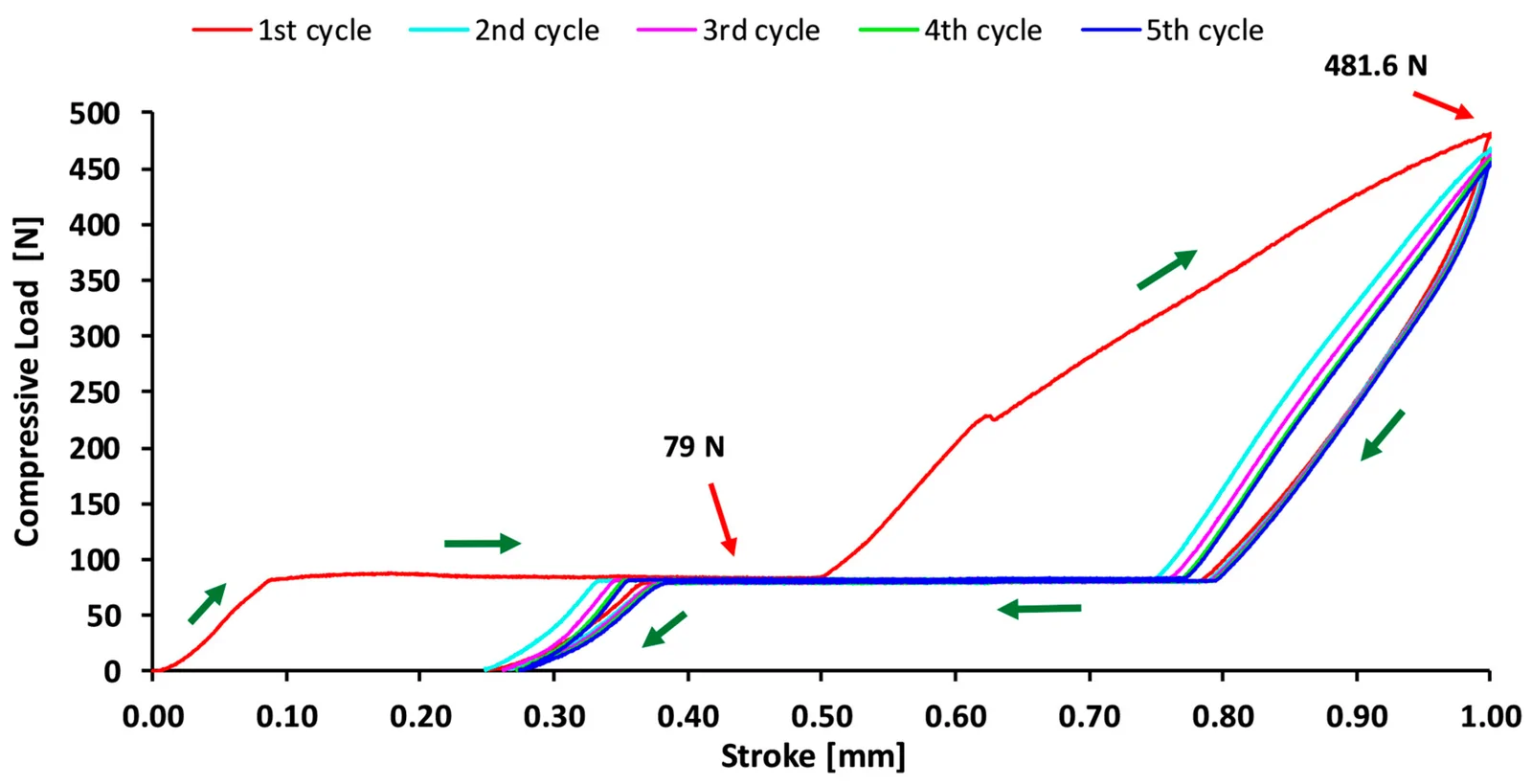
Response of the Fe-Mn-Si-Cr module to five static compression cycles.

The Fe-Mn-Si-Cr module (SCR = 2.29) severely plastic deformed was subjected to five static compression loading–unloading cycles using an INSTRON 3382 testing machine. The tests were performed with a displacement of 1 mm, at a displacement rate of 0.5 mm/min, at room temperature. The force–displacement response of the module during the static compression test is presented in Figure 10.

During the compression test, distinct plateau regions can be observed during both loading and unloading, with the force measured on the plateau varying around ≌79 N. In the first cycle, during loading, the maximum force recorded at a displacement of 1 mm is 481.6 N. During unloading, the path followed by the curve differs from that corresponding to loading, resulting in a well-defined hysteresis loop. This difference reflects the existence of a dissipative component of the mechanical response. In the subsequent cycles, the overall shape of the curves is maintained; however, a pronounced change is observed between the first and second cycles, followed by the progressive overlap of the paths corresponding to the subsequent cycles. This evolution indicates significant mechanical accommodation in the first cycle and a tendency toward stabilization of the cyclic response.

**Figure 11:**
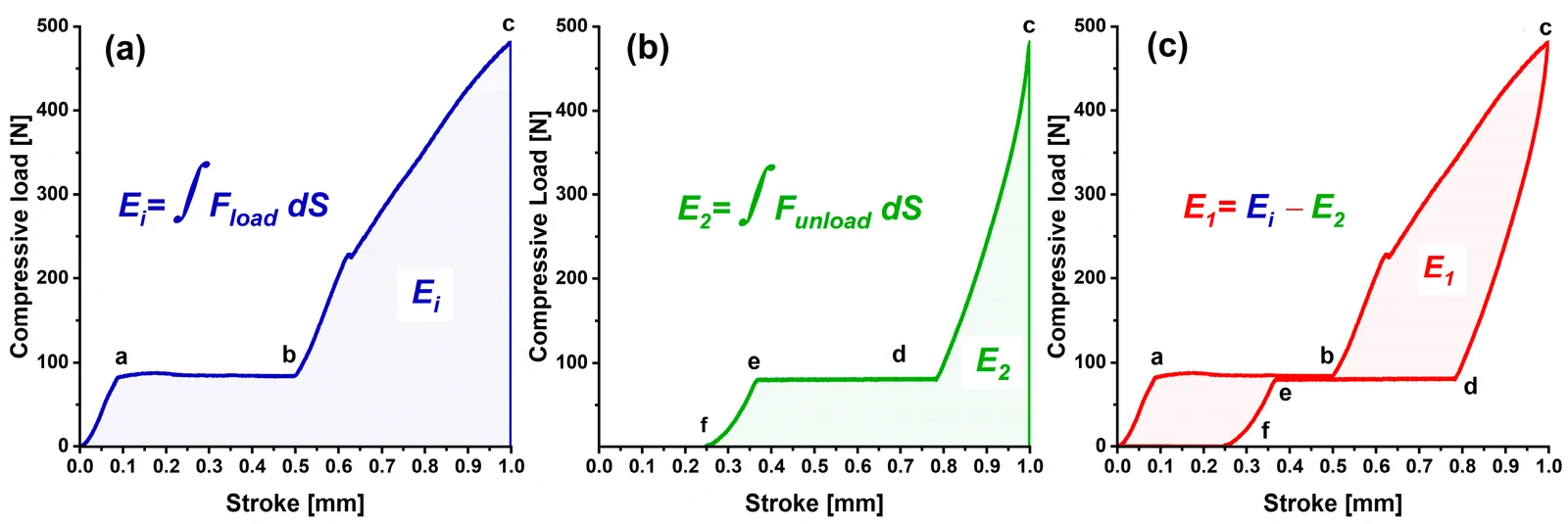
Schematic representation of the area calculation procedure: (**a**) mechanical energy input during loading, *E*_i_; (**b**) energy recovered during unloading, *E*_2_; (**c**) dissipated energy, *E*_1_.

The plateau regions observed during the loading–unloading cycles reflect the complex mechanical response of the Fe-Mn-Si-Cr module. The reversible γ ↔ ε martensitic transformation in Fe-Mn-Si systems is characterized by a broader hysteresis and is closely related to stacking faults and the motion of Shockley partial dislocations. In Fe-Mn-Si-Cr alloys, the shape memory effect is associated with the stress-induced γ (FCC) → ε (HCP) martensitic transformation and the reverse ε → γ transformation upon heating. The force–displacement response presented in Figure 10 exhibits superelastic-like behavior [59,66], because the observed response is influenced not only by the reversible stress-induced martensitic transformation [67] but also by the progressive flattening of the Fe-Mn-Si-Cr module processed by HSHPT. To investigate the superelastic-like behavior of the Fe-Mn-Si-Cr module, the characteristic parameters of the load–displacement curves were determined. Figure 11 illustrates the procedure used to calculate the energy parameters, taking the first loading–unloading cycle of the Fe-Mn-Si-Cr module as an example. The mechanical energies associated with the loading–unloading cycles were calculated by numerical integration (trapezoidal rule) of the corresponding load–displacement curves.

The functional parameters of the module include the energy storage efficiency (*η*) and the recoverable displacement (*S*_rec_). The energy storage efficiency was calculated according to the relationship

(6) $$\eta =\frac{E_{2}}{E_{1}+E_{2}}\cdot 100\;[\%]$$

while the recoverable displacement was determined using the relationship

(7) $$S_{\mathrm{rec}}=\frac{\left(S_{\max}-S_{\mathrm{rem}}\right)}{S_{\max}}\cdot 100\;[\%]$$

where *S*_max_ represents the maximum displacement reached during loading (point c, Figure 11a), whereas *S*_rem_ denotes the residual displacement after unloading (point f, Figure 11b).

**Figure 12:**
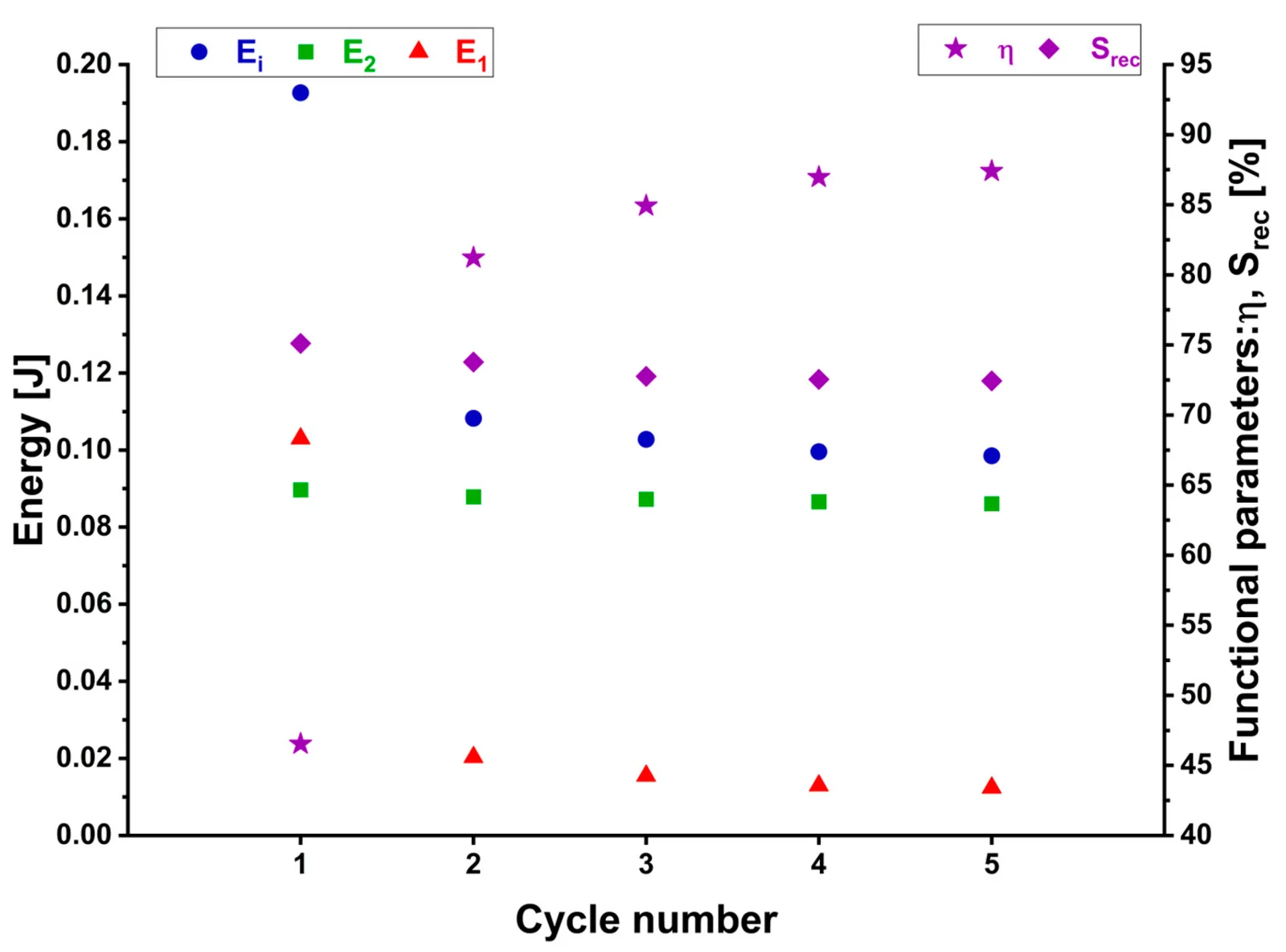
Variation in the energy parameters (energy input during loading, *E*_i_, energy recovered during unloading, *E*_2_, and energy dissipated through hysteresis and internal friction, *E*_1_) and functional parameters (energy storage efficiency, *η*, and recoverable displacement, *S*_rec_) of the Fe-Mn-Si-Cr module.

The evolution of the energy and functional parameters over the five cycles is presented in Figure 12.

**Figure 13:**
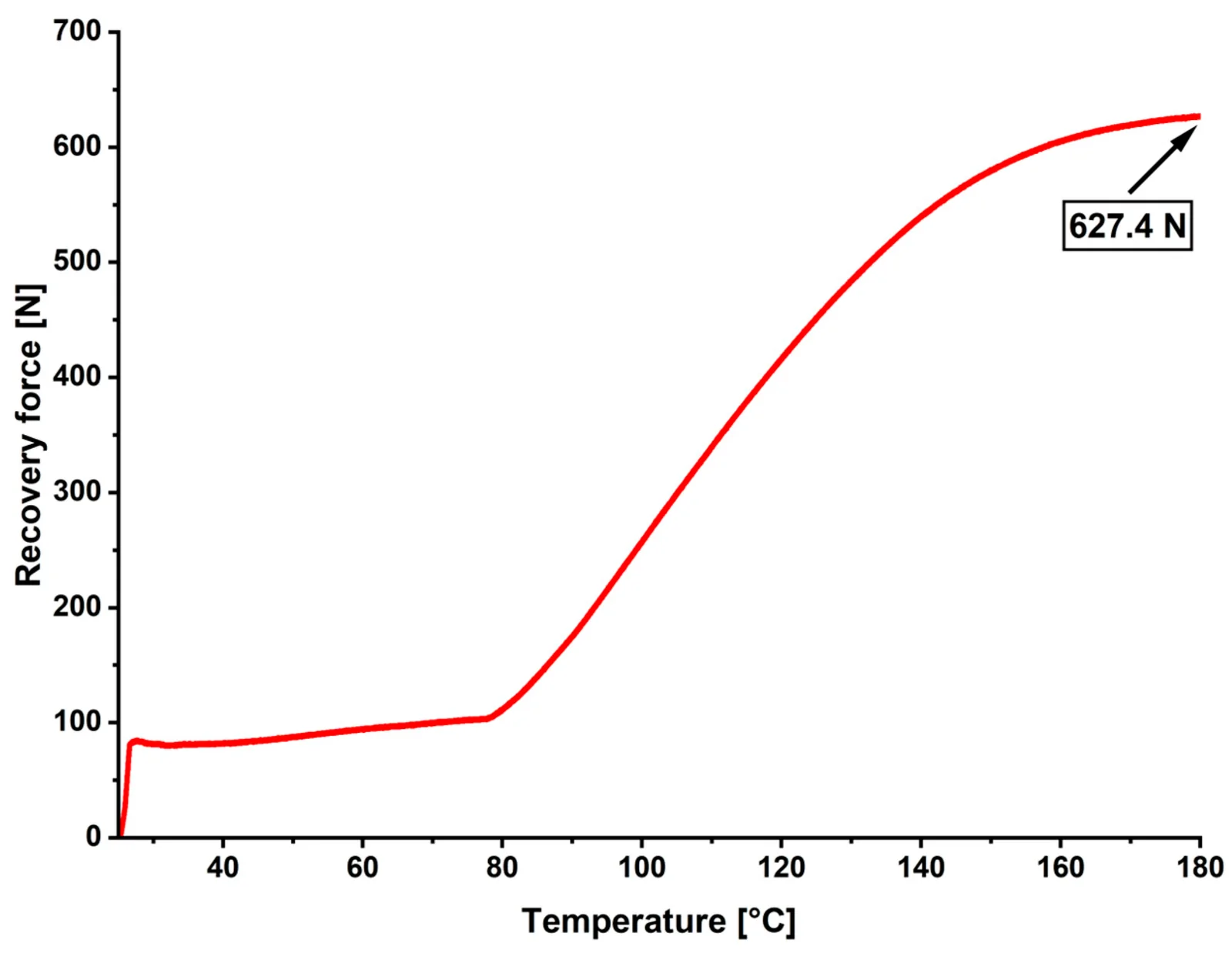
Evolution of the recovery force during heating for the Fe-Mn-Si-Cr module.

The most pronounced change occurs between the first and second cycles. The mechanical energy input *E*_i_ decreases from 0.19271 J in the first cycle to 0.10824 J in the second cycle and then progressively decreases to 0.09852 J in the fifth cycle. In contrast, the recovered energy *E*_2_ varies within a much narrower range, from 0.08969 J to 0.08611 J. Consequently, the dissipated energy *E*_1_ shows the most pronounced decrease: from 0.10301 J in the first cycle to 0.02034 J in the second cycle and subsequently to 0.01241 J in the final cycle. The numerical values thus confirm that the major energetic change occurs in the first cycling interval, after which the response gradually evolves toward a more stable regime. The pronounced reduction in dissipated energy leads to an increase in the energy storage efficiency, *η*, from 46.54% in the first cycle to 81.21% in the second cycle, followed by values of 84.92%, 86.96%, and 87.40% in cycles 3–5. The increase of 34.67 percentage points between the first two cycles is therefore dominant compared with the subsequent variations. This evolution is mainly determined by the reduction in *E*_1_, while *E*_2_ remains comparatively stable. As cycling progresses, an increasingly larger fraction of the input mechanical energy is returned during unloading, whereas the dissipated fraction decreases. The recoverable displacement *S*_rec_ exhibits a different and much more gradual evolution. The value decreases from 75.11% in the first cycle to 73.78% in the second cycle and then to 72.76%, 72.55%, and 72.43% in cycles 3–5. The total decrease is only 2.68 percentage points, and the difference between cycles 4 and 5 is approximately 0.12 percentage points. This trend, together with the progressive reduction in *E*_1_ and the increasingly pronounced overlap of the force–displacement curves, indicates a cyclic accommodation process of the module and an approach toward a repeatable response after the first cycles. Energy efficiency and dimensional recovery do not evolve identically. While *η* increases by 40.86 percentage points between cycles 1 and 5 as a result of the strong reduction in energy losses, *S*_rec_ decreases moderately from 75.11% to 72.43%. Therefore, the increase in energy efficiency during cycling should not be interpreted as an increase in displacement recovery, but rather as the result of the progressive reduction in hysteresis and dissipative processes. At the same time, the stabilization of the *S*_rec_ values in the final cycles shows that dimensional recovery tends toward an almost constant level. Although complete stabilization was not achieved over five cycles, the observed trends indicate the onset of a stable operating regime. The progressive decrease in dissipated energy (*E*_1_) increased the energy storage efficiency (*η*), while the decreasing variation in recoverable displacement (*S*_rec_) suggests convergence toward an equilibrium value. This behavior is characteristic of the mechanical training of shape memory alloys and reflects the stabilization of stress-induced martensitic transformations and the pseudoelastic response of the modules after HSHPT. The shape memory effect under constrained recovery conditions was evaluated using a second mechanical protocol. The Fe-Mn-Si-Cr module was partially compressed to a constant displacement of 0.4 mm between flat, unlubricated surfaces and maintained in the deformed state. Subsequently, the specimen was heated from room temperature to 180 °C in the thermal chamber of the testing machine to evaluate the recovery force generated during return to the initial shape. The resulting force–temperature response of the Fe-Mn-Si-Cr module is presented in Figure 13. The increase in force during heating demonstrates activation of the shape memory effect under constrained recovery conditions. In the Fe-Mn-Si-Cr alloy, mechanical deformation promotes the γ → ε martensitic transformation, and part of the stress-induced ε-martensite may persist after the stress is reduced as a consequence of the hysteresis characteristic of this transformation.

Heating promotes the reverse ε → γ transformation, which constitutes the fundamental mechanism of shape recovery in this class of alloys [42,67]. Because the module displacement is maintained constant at 0.4 mm, the free dimensional recovery associated with the reverse transformation is prevented, and the recovery tendency manifests itself through the development of an axial recovery force. The maximum value of 627.4 N at 180 °C highlights the ability of the HSHPT-processed Fe-Mn-Si-Cr module to convert thermally activated recovery into a functional mechanical response under constrained conditions.

Overall, the two mechanical protocols highlight two complementary components of the functional behavior of the Fe-Mn-Si-Cr module. Cyclic testing at room temperature indicates pronounced accommodation in the first cycle, reflected by the decrease in dissipated energy *E*_1_ from 0.10301 to 0.01241 J and the increase in energy storage efficiency from 46.54 to 87.40%, while *S*_rec_ decreases moderately from 75.11 to 72.43%. These evolutions describe the progressive stabilization of the cyclic response without implying a concomitant increase in dimensional recovery. Testing under constrained recovery conditions reveals a distinct manifestation of the shape memory effect: the thermally activated reverse ε → γ transformation generates a recovery force that reaches 627.4 N at 180 °C.

## 4. Conclusions

In this study, a multiscale characterization was performed on a truncated-conical module made of the Fe57Mn27Si11Cr5 (% at.) shape memory alloy, directly obtained by HSHPT processing at an estimated logarithmic strain of *ε* = 1.10. The results highlight the pronounced effect of severe plastic deformation on the microstructure, crystalline state, and functional behavior of the module.

The main conclusions of this study are as follows:

1. HSHPT processing eliminated the dendritic structure characteristic of the initial material and produced a severely deformed and refined microstructure, characterized by deformation bands and fibrous regions oriented along the material flow direction. SEM investigations revealed lamellar regions and two preferred orientations of the ε-martensite variants.
2. XRD analysis confirmed that γ-FCC austenite remained the dominant crystalline phase after processing. The main γ(111) reflection shifted from 2θ = 43.47° for the alloy in the as-received state to 43.12° for the HSHPT module, corresponding to an increase in the interplanar spacing and in the lattice parameter from approximately 3.60 to 3.63 Å. The mean coherent diffraction domain size, *τ*, estimated using the Scherrer equation, increased from 12.93 to 20.02 nm for the γ (111) reflection. These results indicate measurable changes in the crystal lattice state induced by HSHPT.
3. AFM characterization revealed a surface with nanoscale roughness, characterized by an arithmetic mean roughness (*R*_a_) of 14.59 nm and a root mean square roughness (*R*_q_) of 19.77 nm, together with an asymmetric height distribution. The Flooding procedure enabled the identification of 677 grains over the scanned area of 40 × 40 µm^2^. The ECD distribution is predominantly submicrometric, with 89.7% of the quantified grains having ECD values below 400 nm and a mean value of 253.39 nm. The accumulation of measurements near the lower quantification limit of 176.31 nm is also compatible with the presence of a population of nanoscale grains that cannot be individually quantified under the scanning conditions used.
4. Mechanical tests demonstrated stable functional behavior and progressive cyclic stabilization. The module exhibited a superelastic-like load–displacement response, accompanied by an increase in energy storage efficiency and a decrease in dissipated energy during repeated compression cycles. Under constrained recovery conditions between flat surfaces, after being compressed to a displacement of 0.4 mm and heated to 180 °C, the module developed a maximum recovery force of 627.4 N.

The present study was based on OM, SEM, XRD, AFM, and mechanical testing. While these techniques provide valuable insights into microstructural refinement and functional performance, the direct characterization of stacking faults, dislocation arrangements, grain-boundary misorientations, and nanoscale phase morphology requires complementary techniques such as TEM and EBSD. In addition, owing to the torsional nature of deformation and the truncated conical geometry employed in HSHPT processing, possible local variations in strain distribution were not investigated in the present work. Therefore, future studies will focus on advanced microstructural characterization, as well as on the evaluation of strain heterogeneity and its influence on microstructural evolution and functional properties.

Overall, the results demonstrate that HSHPT processing enables the direct fabrication of a functional Fe-Mn-Si-Cr module with a predefined geometry, in which microstructural refinement, structural changes, and cyclic accommodation coexist with the preservation of an active shape memory response. Correlating OM, SEM, XRD, and AFM investigations with mechanical testing provides an integrated characterization of the state obtained after HSHPT and supports the use of this technology for the further development of active elements and composite systems based on shape memory alloys for applications in self-adaptive bearing preload systems and other smart-actuator applications.

## Figures and Tables

**Table 1:** Geometrical parameters of Fe-Mn-Si-Cr module.

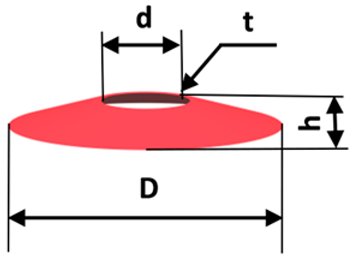	***D*** **, mm**	***d*** **, mm**	***h*** **, mm**	***t*** **, mm**	$\mathrm{SCR}=\frac{h-t}{t}$
	25.24	8.20	2.30	0.70	2.29

**Table 2:** Structural parameters determined from Voigt fitting of the γ (111) reflection.

Sample	Phase	hkl	2θ [°]	Voigt FWHM [°]	*d*_111_ [Å]	*a*_FCC_ [Å]	Scherrer τ [nm]	Adjusted *R*^2^
Fe-Mn-Si-Cr as-received	γ-FCC	(111)	43.47	0.69	2.08	3.60	12.93	0.998
Fe-Mn-Si-Cr module	γ-FCC	(111)	43.12	0.45	2.10	3.63	20.02	0.990

**Table 3:** Topographic and roughness parameters determined by AFM.

Roughness Parameter	Value
RMS roughness, *R*_q_ [nm]	19.7678
Minimum value [nm]	−81.9321
Maximum value [nm]	168.9383
Peak-to-peak [nm]	250.8704
Roughness average, *R*_a_ [nm]	14.5948
Average height [nm]	0.0000
Surface skewness, S_sk_	0.8028
Surface kurtosis, S_ku_	6.0782
Plane offset [nm]	31.5521
Ironed surface [µm^2^]	1608.2505

## Data Availability

Data supporting the results of this study are available from the corresponding author upon reasonable request.

## Abbreviations

The following abbreviations are used in this manuscript:

2D	Two-dimensional
3D	Three-dimensional
AFM	Atomic force microscopy
ECD	Equivalent Circular Diameter
FCC	Face-centered cubic
FWHM	Full Width at Half Maximum
HCP	Hexagonal close-packed
HPT	High-Pressure Torsion
HSHPT	High-Speed High-Pressure Torsion
OM	Optical microscopy
RMS	Root-mean-square
SCR	Shape ratio coefficient
SEM	Scanning electron microscopy
SFE	Stacking-fault energy
SMA	Shape memory alloy
SME	Shape memory effect
SPD	Severe plastic deformation
UFG	Ultrafine-grained
XRD	X-ray diffraction

## References

[1] Sato A., Chishima E., Soma K., Mori T. (1982). Shape memory effect in γ⇄ϵ transformation in Fe-30Mn-1Si alloy single crystals. Acta Metall..

[2] Sato A., Chishima E., Yamaji Y., Mori T. (1984). Orientation and composition dependencies of shape memory effect IN Fe-Mn-Si alloys. Acta Metall..

[3] Yang J.H., Chen H., Wayman C.M. (1992). Development of Fe-based shape memory alloys associated with face-centered cubic-hexagonal close-packed martensitic transformations: Part I. shape memory behavior. Metall. Trans. A.

[4] Gu Q., VAN Humbeeck J., Delaey L. (1994). A review on the martensitic transformation and shape memory effect in Fe-Mn-Si alloys. J. Phys. IV.

[5] Kajiwara S. (1999). Characteristic features of shape memory effect and related transformation behavior in Fe-based alloys. Mater. Sci. Eng. A.

[6] Otsuka H., Yamada H., Maruyama T., Tanahashi H., Matsuda S., Murakami M. (1990). Effects of alloying additions on Fe-Mn-Si shape memory alloys. ISIJ Int..

[7] Stanford N.E., Chen K., Dunne D.P., Jin X.-J. (2007). Effect of Alloying Additions on the SFE, Neel Temperature and Shape Memory Effect in Fe-Mn-Si-based Alloys. ISIJ Int..

[8] Stanford N., Dunne D. (2010). Effect of Si on the reversibility of stress-induced martensite in Fe–Mn–Si shape memory alloys. Acta Mater..

[9] Cotes S., Guillermet A., Sade M. (1998). Phase stability and fcc/hcp martensitic transformation in Fe–Mn–Si alloys. J. Alloy. Compd..

[10] Guerrero L., La Roca P., Malamud F., Baruj A., Sade M. (2017). Composition effects on the fcc-hcp martensitic transformation and on the magnetic ordering of the fcc structure in Fe-Mn-Cr alloys. Mater. Des..

[11] Pan M.-M., Zhang X.-M., Zhou D., Misra R., Chen P., Su X.-B. (2020). On the significance of C and Co on shape memory performance of Fe–Mn–Si–Cr–Ni shape memory alloy. Mater. Sci. Eng. A.

[12] Kustov B., Lähderanta E., Van Humbeeck J., Serra J.T. (2024). Reverse ε→γ transformations of temperature-induced and stress-induced martensites in Fe–Mn–Si alloys with shape memory effect. J. Mater. Res. Technol..

[13] Min X., Sawaguchi T., Zhang X., Tsuzaki K. (2012). Reasons for incomplete shape recovery in polycrystalline Fe–Mn–Si shape memory alloys. Scr. Mater..

[14] Sawaguchi T., Bujoreanu L.-G., Kikuchi T., Ogawa K., Koyama M., Murakami M. (2008). Mechanism of reversible transformation-induced plasticity of Fe–Mn–Si shape memory alloys. Scr. Mater..

[15] Maji B.C., Krishnan M. (2003). The effect of microstructure on the shape recovery of a Fe–Mn–Si–Cr–Ni stainless steel shape memory alloy. Scr. Mater..

[16] Wen Y., Peng H., Raabe D., Gutierrez-Urrutia I., Chen J., Du Y. (2014). Large recovery strain in Fe-Mn-Si-based shape memory steels obtained by engineering annealing twin boundaries. Nat. Commun..

[17] Wen Y., Zhang W., Li N., Peng H., Xiong L. (2007). Principle and realization of improving shape memory effect in Fe–Mn–Si–Cr–Ni alloy through aligned precipitations of second-phase particles. Acta Mater..

[18] Dong Z., Klotz U.E., Leinenbach C., Bergamini A., Czaderski C., Motavalli M. (2009). A Novel Fe-Mn-Si Shape Memory Alloy With Improved Shape Recovery Properties by VC Precipitation. Adv. Eng. Mater..

[19] Kajiwara S., Liu D., Kikuchi T., Shinya N. (2001). Remarkable improvement of shape memory effect in Fe-Mn-Si based shape memory alloys by producing NbC precipitates. Scr. Mater..

[20] Stanford N., Dunne D. (2007). Effect of second-phase particles on shape memory in Fe–Mn–Si-based alloys. Mater. Sci. Eng. A.

[21] Lai M., Li Y., Lillpopp L., Ponge D., Will S., Raabe D. (2018). On the origin of the improvement of shape memory effect by precipitating VC in Fe–Mn–Si-based shape memory alloys. Acta Mater..

[22] Stanford N., Dunne D., Li H. (2008). Re-examination of the effect of NbC precipitation on shape memory in Fe–Mn–Si-based alloys. Scr. Mater..

[23] Stanford N., Dunne D. (2006). Thermo-mechanical processing and the shape memory effect in an Fe–Mn–Si-based shape memory alloy. Mater. Sci. Eng. A.

[24] Peng H., Wen Y., Xiong L., Li N. (2008). Influence of initial microstructures on effectiveness of training in a FeMnSiCrNi shape memory alloy. Mater. Sci. Eng. A.

[25] Collazo A., Figueroa R., Mariño-Martínez C., Pérez C. (2021). Microstructure and Thermomechanical Characterization of Fe-28Mn-6Si-5Cr Shape Memory Alloy. Metals.

[26] Wang D., Liu D., Dong Z., Liu W., Chen J. (2001). Improvement of shape memory effect by ausforming in Fe–28Mn–6Si–5Cr alloy. Mater. Sci. Eng. A.

[27] Liu D.Z., Kajiwara S., Kikuchi T., Shinya N. (2003). Atomic force microscopy study on microstructural changes by “training” in Fe-Mn-Si-based shape memory alloys. Philos. Mag..

[28] Park K., Choi E., Kim W. (2022). Grain size effect on the recovery stress and strain of a Fe–Mn–Si shape memory alloy in a wide range of grain sizes. Mater. Sci. Eng. A.

[29] Wang G., Peng H., Zhang C., Wang S., Wen Y. (2016). Relationship among grain size, annealing twins and shape memory effect in Fe–Mn–Si based shape memory alloys. Smart Mater. Struct..

[30] Zhang W., Li N., Wen Y.H., Huang S.K. (2010). Grain refinement and carbide precipitation in Fe–Mn–Si–Cr–Ni–C shape memory alloy fabricated by equal channel angular pressing. Mater. Sci. Technol..

[31] Valiev R.Z., Islamgaliev R.K., Alexandrov I.V. (2000). Bulk nanostructured materials from severe plastic deformation. Prog. Mater. Sci..

[32] Edalati K., Bachmaier A., Beloshenko V.A., Beygelzimer Y., Blank V.D., Botta W.J., Bryła K., Čížek J., Divinski S., Enikeev N.A. (2022). Nanomaterials by severe plastic deformation: Review of historical developments and recent advances. Mater. Res. Lett..

[33] Ebrahimi M., Attarilar S., Gode C., Kandavalli S.R., Shamsborhan M., Wang Q. (2023). Conceptual Analysis on Severe Plastic Deformation Processes of Shape Memory Alloys: Mechanical Properties and Microstructure Characterization. Metals.

[34] Zhilyaev A.P., Langdon T.G. (2008). Using high-pressure torsion for metal processing: Fundamentals and applications. Prog. Mater. Sci..

[35] Gurău G., Gurău C., Potecaşu O., Alexandru P., Bujoreanu L.-G. (2014). Novel High-Speed High Pressure Torsion Technology for Obtaining Fe-Mn-Si-Cr Shape Memory Alloy Active Elements. J. Mater. Eng. Perform..

[36] Gurau G., Gurau C., Sampath V., Bujoreanu L.G. (2016). Investigations of a nanostructured FeMnSi shape memory alloy produced via severe plastic deformation. Int. J. Miner. Metall. Mater..

[37] Bujoreanu L.-G., Goanță V., Cimpoeșu N., Gurău C., Suru M.-G., Mihalache E., Gurău G. (2015). Hardness-gradient reversion in FeMnSiCr shape memory alloy modules produced by high-speed high pressure torsion. MATEC Web Conf..

[38] Gurau C., Gurau G., Bujoreanu L., Fernandes F. (2015). A Comparative Study of Austenitic Structure in NiTi and Fe Based Shape Memory Alloys after Severe Plastic Deformation. Mater. Today Proc..

[39] GGurau G., Gurau C., Banu M., Bujoreanu L.G. (2017). Microstructural Evolution in Ultrafine Grained FeMnSiCr Shape Memory Alloy Modules. Adv. Mater. Res..

[40] Lee W., Weber B., Feltrin G., Czaderski C., Motavalli M., Leinenbach C. (2013). Phase transformation behavior under uniaxial deformation of an Fe–Mn–Si–Cr–Ni–VC shape memory alloy. Mater. Sci. Eng. A.

[41] Lee W.J., Weber B., Feltrin G., Czaderski C., Motavalli M., Leinenbach C. (2013). Stress recovery behaviour of an Fe–Mn–Si–Cr–Ni–VC shape memory alloy used for prestressing. Smart Mater. Struct..

[42] Leinenbach C., Arabi-Hashemi A., Lee W., Lis A., Sadegh-Ahmadi M., Van Petegem S., Panzner T., Van Swygenhoven H. (2017). Characterization of the deformation and phase transformation behavior of VC-free and VC-containing FeMnSi-based shape memory alloys by in situ neutron diffraction. Mater. Sci. Eng. A.

[43] Dias D., Nakamatsu S., Della Rovere C.A., Otubo J., Mariano N.A. (2019). Characterization and Corrosion Resistance Behavior of Shape Memory Stainless Steel Developed by Alternate Routes. Metals.

[44] Hong K.N., Lee S.G., Han S.H., Yeon Y.M. (2018). Evaluation of Fe-Based Shape Memory Alloy (Fe-SMA) as Strengthening Material for Reinforced Concrete Structures. Appl. Sci..

[45] Cladera A., Weber B., Leinenbach C., Czaderski C., Shahverdi M., Motavalli M. (2014). Iron-based shape memory alloys for civil engineering structures: An overview. Constr. Build. Mater..

[46] Qiang X., Wu Y., Wang Y., Jiang X. (2023). Research Progress and Applications of Fe-Mn-Si-Based Shape Memory Alloys on Reinforcing Steel and Concrete Bridges. Appl. Sci..

[47] Sawaguchi T., Sahu P., Kikuchi T., Ogawa K., Kajiwara S., Kushibe A., Higashino M., Ogawa T. (2006). Vibration mitigation by the reversible fcc/hcp martensitic transformation during cyclic tension–compression loading of an Fe–Mn–Si-based shape memory alloy. Scr. Mater..

[48] Ferretto I., Kim D., Lee W., Hosseini E., della Ventura N., Sharma A., Sofras C., Capek J., Polatidis E., Leinenbach C. (2023). Shape memory and mechanical properties of a Fe-Mn-Si-based shape memory alloy: Effect of crystallographic texture generated during additive manufacturing. Mater. Des..

[49] Leinenbach C., Kramer H., Bernhard C., Eifler D. (2012). Thermo-Mechanical Properties of an Fe–Mn–Si–Cr–Ni–VC Shape Memory Alloy with Low Transformation Temperature. Adv. Eng. Mater..

[50] Wang W., Wang X. (2020). Tests, model, and applications for coned-disc-spring vertical isolation bearings. Bull. Earthq. Eng..

[51] Gurau G., Gurau C., Bujoreanu L.G., Sampath V. (2017). A Versatile Method for Nanostructuring Metals, Alloys and Metal Based Composites. IOP Conf. Ser. Mater. Sci. Eng..

[52] Horcas I., Fernández R., Gómez-Rodriguez J.M., Colchero J., Gomez-Herrero J., Baro A.M. (2007). WSXM: A software for scanning probe microscopy and a tool for nanotechnology. Rev. Sci. Instrum..

[53] Valiev R.Z., Estrin Y., Horita Z., Langdon T.G., Zechetbauer M.J., Zhu Y.T. (2006). Producing bulk ultrafine-grained materials by severe plastic deformation. JOM.

[54] Gurau C., Gurau G., Tolea F., Popescu B., Banu M., Bujoreanu L.-G. (2021). The Effect of the In-Situ Heat Treatment on the Martensitic Transformation and Specific Properties of the Fe-Mn-Si-Cr Shape Memory Alloys Processed by HSHPT Severe Plastic Deformation. Materials.

[55] Estrin Y., Vinogradov A. (2013). Extreme grain refinement by severe plastic deformation: A wealth of challenging science. Acta Mater..

[56] Sauvage X., Wilde G., Divinski S.V., Horita Z., Valiev R.Z. (2012). Grain boundaries in ultrafine grained materials processed by severe plastic deformation and related phenomena. Mater. Sci. Eng. A.

[57] Rybalchenko O., Martynenko N., Anisimova N., Rybalchenko G., Tabachkova N., Lukyanova E., Shchetinin I., Temralieva D., Tokar A., Straumal P. (2025). Relationship Between Structure and Functional Properties of Ultrafine-Grained Fe-Mn-Si Alloys for Temporary Implants. Crystals.

[58] Del-Río L., Nó M.L., Gómez R., García-Sesma L., Urionabarrenetxea E., Ortega P., Mancisidor A.M., Sebastian M.S., Burgos N., Juan J.M.S. (2023). Additive Manufacturing of Fe-Mn-Si-Based Shape Memory Alloys: State of the Art, Challenges and Opportunities. Materials.

[59] Arabi-Hashemi A., Lee W., Leinenbach C. (2018). Recovery stress formation in FeMnSi based shape memory alloys: Impact of precipitates, texture and grain size. Mater. Des..

[60] Langford J.I., Wilson A.J.C. (1978). Scherrer after sixty years: A survey and some new results in the determination of crystallite size. J. Appl. Crystallogr..

[61] Gesing T.M., Robben L. (2024). Determination of the average crystallite size and the crystallite size distribution: The envelope function approach EnvACS. J. Appl. Crystallogr..

[62] Akinyemi A.F., Agboola O., Alagbe E.E., Sadiku R. (2026). Investigation of microbial adhesion and biofilm formation potential on biodegradable PLA-based TiO_2_/Chitosan nanocomposite membranes in relation to surface morphology. Polymer.

[63] Oliver R.A., Kappers M.J., Humphreys C.J. (2006). Insights into the origin of threading dislocations in GaN∕Al_2_O_3_ from atomic force microscopy. Appl. Phys. Lett..

[64] Wünsche J., Tarabella G., Bertolazzi S., Bocoum M., Coppedè N., Barba L., Arrighetti G., Lutterotti L., Iannotta S., Cicoira F. (2013). The correlation between gate dielectric, film growth, and charge transport in organic thin film transistors: The case of vacuum-sublimed tetracene thin films. J. Mater. Chem. C.

[65] Okunev A.G., Mashukov M.Y., Nartova A.V., Matveev A.V. (2020). Nanoparticle Recognition on Scanning Probe Microscopy Images Using Computer Vision and Deep Learning. Nanomaterials.

[66] Van Caenegem N., Duprez L., Verbeken K., Segers D., Houbaert Y. (2008). Stresses related to the shape memory effect in Fe–Mn–Si-based shape memory alloys. Mater. Sci. Eng. A.

[67] Bergeon N., Guenin G., Esnouf C. (1998). Microstructural analysis of the stress-induced ε martensite in a Fe–Mn–Si–Cr–Ni shape memory alloy. Mater. Sci. Eng. A.

